# Colonoscopy-Based Diagnosis of *Dibothriocephalus nihonkaiensis* Infection Protruding into the Ascending Colon

**DOI:** 10.4269/ajtmh.25-0254

**Published:** 2025-07-29

**Authors:** Eisuke Adachi, Hiroyuki Nagai, Makoto Saito, Akihiro Osawa

**Affiliations:** ^1^ Department of Infectious Diseases and Applied Immunology, IMSUT Hospital of The Institute of Medical Science, The University of Tokyo, Tokyo, Japan;; ^2^ Osawa Gastro Entero Proctology & Endoscopy Clinic Shinagawa, Tokyo, Japan

## Abstract

We report two recent cases of diphyllobothriid cestode infection identified during colonoscopy in asymptomatic individuals in Tokyo, Japan. Both patients lacked distinctive dietary habits, consuming only commonly available raw fish. In one case, the tapeworm was found in the terminal ileum; in the other case, the parasite extended into the ascending colon, which is a rare site of detection during colonoscopy. Morphological features were consistent with *Dibothriocephalus nihonkaiensis*. Blood tests revealed no notable abnormalities. These cases underscore that such infections may occur even in individuals without high-risk dietary behaviors. With increased use of colonoscopy and the global consumption of raw fish, similar detections may become more common, emphasizing the need for clinical awareness, even in patients without recognized dietary risk factors.

## INTRODUCTION

Human infection with *Dibothriocephalus* spp. is primarily caused by consumption of raw or undercooked fish harboring plerocercoid larvae. In Japan, *Dibothriocephalus nihonkaiensis* accounts for the majority of reported cases.^1^ This tapeworm species is endemic to the region and is typically acquired through the ingestion of marine fish, such as salmon. We report two recent cases of diphyllobothriid cestode infection in Tokyo, Japan that were identified through colonoscopic findings in patients without apparent high-risk dietary habits. These cases underscore the potential for incidental parasitic detection during colonoscopy, even in asymptomatic individuals and those consuming only commonly available raw fish.

## CASE REPORT

A 32-year-old Japanese man living in Tokyo spontaneously excreted a 10-cm tapeworm into the toilet. Subsequent colonoscopy at a local clinic revealed the presence of a tapeworm located in the terminal ileum. The patient was otherwise asymptomatic and reported only occasional consumption of sushi, mainly from chain-style restaurants that primarily serve frozen, mass-distributed seafood products.

**Figure 1.**
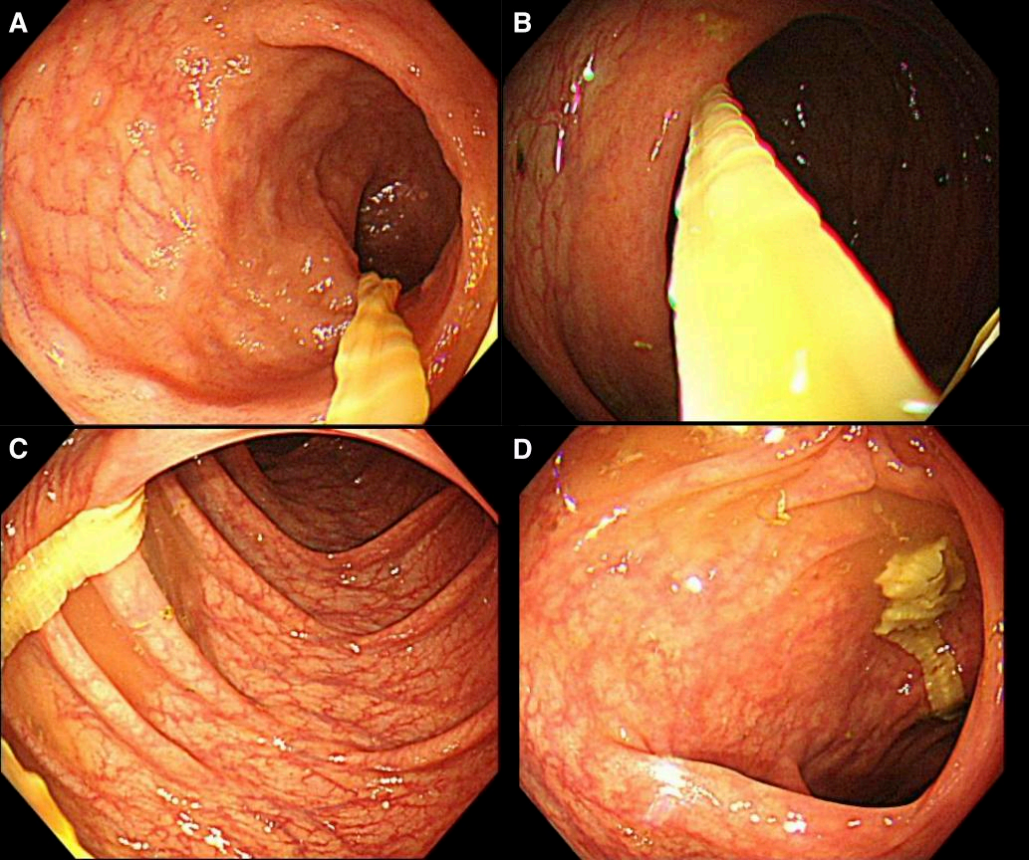
Endoscopic findings of *Dibothriocephalus nihonkaiensis* infection. (**A** and **B**) Images of the ileum showing the tapeworm extending proximally. (**C** and **D**) Images of the ascending colon showing the tapeworm protruding from the terminal ileum into the colonic lumen.

Similarly, a 44-year-old Japanese woman residing in Tokyo passed a tapeworm without accompanying symptoms. Endoscopic evaluation revealed a white, ribbon-like tapeworm protruding from the ileum into the ascending colon (Figure 1). Her dietary history included infrequent consumption of raw salmon, mainly at general restaurants or through standard commercial sources, without any distinctive culinary preferences.

Both patients were referred to our clinic for further evaluation and management. Blood test results in both cases revealed no remarkable abnormalities. After administration of praziquantel and bowel lavage, complete elimination of the parasites was achieved, including retrieval of the scolex, which was confirmed in the discharged tapeworm in both cases. Morphological examination identified the tapeworms as consistent with *Dibothriocephalus* spp. However, species-level identification was not possible based on morphology alone. Notably, neither patient had any history of consuming raw freshwater fish or any history of residence in or travel to regions where *Dibothriocephalus latus* is endemic, such as North America or parts of Europe; this allowed us to reasonably conclude that the causative organism was *D. nihonkaiensis*, a marine species endemic to Japan that is commonly acquired through ingestion of raw or undercooked marine fish.^2^

## DISCUSSION

Although *D. nihonkaiensis* is known to inhabit the small intestine, it is generally considered challenging to detect through endoscopy because of its anatomical location.^3^ Capsule endoscopy, which allows visualization of the small intestine, can readily detect *D. nihonkaiensis*.^4^ In contrast, colonoscopy generally does not visualize the small intestine beyond the terminal ileum. Therefore, the detection of *D. nihonkaiensis* during colonoscopy remains uncommon. These two cases provide visual documentation of the parasite, which was observed in the ileum in one case and extending into the ascending colon in the other case, highlighting the importance of maintaining clinical awareness even in the absence of gastrointestinal symptoms.

In Japan, eating raw fish is deeply rooted in culinary culture. Freshly caught, unfrozen marine fish, particularly those served at certain sushi restaurants, have traditionally been considered a major source of *Dibothriocephalus* spp. infections. These infections have long been associated with individuals who actively seek out sashimi that has not undergone freezing. However, our cases demonstrate that infection can also occur in individuals who dine at ordinary restaurants and mass-market sushi establishments that rely on frozen seafood. This suggests that the risk of infection may extend beyond previously assumed boundaries. Moreover, tapeworm infections have also been reported in European countries, where freezing of fish intended for raw consumption is legally mandated. These reports indicate that transmission can still occur despite adherence to regulatory standards designed to reduce parasitic risk.

Although *D. nihonkaiensis* infections are relatively rare and typically asymptomatic except for the passage of proglottids, they are not associated with vitamin B12 deficiency or megaloblastic anemia, which are characteristic features of infections caused by *D. latus*.^5^ Eosinophilia and other hematological abnormalities that can be seen in parasitic infections are rare in *Dibothriocephalus* spp. The incidental passage of tapeworm segments remains the most common reason for clinical presentation. Given the increasing global popularity of sushi and the widespread use of gastrointestinal endoscopy, these findings underscore the need for heightened awareness of tapeworm infections, even in low-risk populations and nonendemic settings.^2^

*Dibothriocephalus nihonkaiensis* infections are typically associated with the consumption of raw or undercooked marine fish, whereas *D. latus* is more commonly linked to freshwater fish. Although genetic testing is required for definitive species-level identification, such testing is not routinely performed in clinical practice. However, most cases of *Dibothriocephalus* spp. reported in Japan are epidemiologically attributed to *D. nihonkaiensis*.^1^ Therefore, in the absence of freshwater fish consumption or any history of residence in or travel to regions where *D. latus* is endemic, such as North America or parts of Europe, *Dibothriocephalus* spp. infections identified in Japan are epidemiologically presumed to be caused by *D. nihonkaiensis*.

In addition to *D nihonkaiensis*, other tapeworms that can use marine organisms as intermediate hosts include *Spirometra erinaceieuropaei*, *Diplogonoporus balaenopterae*, and *Adenocephalus pacificus*. Apart from *D. latus*, tapeworms that use freshwater fish as intermediate hosts include *Dibothriocephalus dendriticus*, *Schyzocotyle acheilognathi*, and *Dibothriocephalus stemmacephalus*. However, human infections caused by species other than *D. nihonkaiensis* and *D. latus* are extremely rare.^6^^,^^7^ It is conceivable that the persistence of *D. nihonkaiensis* infections in Japan, albeit rare, may be partially attributable to cultural preferences for consuming raw fish that has not undergone freezing. This practice is often regarded as a hallmark of freshness and quality in traditional Japanese cuisine. Although freezing fish is an effective method for preventing parasitic infections, it is not required by Japanese law. Modifying such deeply ingrained culinary traditions is likely to be difficult. As observed in many regionally endemic infectious diseases, the relationship between local dietary customs and infection risk is a critical factor that must be acknowledged.^8^^,^^9^

## CONCLUSION

With the growing global popularity of sushi and the increasing availability and routine use of gastrointestinal endoscopy, including its incorporation into general health checkups, it is likely that similar cases of diphyllobothriid cestode infection will be more frequently identified, not only in Japan but also internationally, particularly because such infections can occur even in asymptomatic individuals without any distinctive dietary habits.^10^^,^^11^ Greater recognition of these incidental findings may contribute to earlier diagnosis, a more accurate understanding of epidemiological patterns, and improved patient education regarding foodborne parasitic risks.
